# SLRProp: A Back-Propagation Variant of Sparse Low Rank Method for DNNs Reduction

**DOI:** 10.3390/s23052718

**Published:** 2023-03-02

**Authors:** Asier Garmendia-Orbegozo, Jose David Nuñez-Gonzalez, Miguel Angel Anton

**Affiliations:** 1 Department of Applied Mathematics, University of the Basque Country UPV/EHU, 20600 Eibar, Spain; 2 TECNALIA, Basque Research and Technology Alliance (BRTA), 20009 San Sebastian, Spain

**Keywords:** pruning, deep learning, edge computing

## Abstract

Application of deep neural networks (DNN) in edge computing has emerged as a consequence of the need of real time and distributed response of different devices in a large number of scenarios. To this end, shredding these original structures is urgent due to the high number of parameters needed to represent them. As a consequence, the most representative components of different layers are kept in order to maintain the network’s accuracy as close as possible to the entire network’s ones. To do so, two different approaches have been developed in this work. First, the Sparse Low Rank Method (SLR) has been applied to two different Fully Connected (FC) layers to watch their effect on the final response, and the method has been applied to the latest of these layers as a duplicate. On the contrary, SLRProp has been proposed as a variant case, where the relevances of the previous FC layer’s components were weighed as the sum of the products of each of these neurons’ absolute values and the relevances of the neurons from the last FC layer that are connected with the neurons from the previous FC layer. Thus, the relationship of relevances across layer was considered. Experiments have been carried out in well-known architectures to conclude whether the relevances throughout layers have less effect on the final response of the network than the independent relevances intra-layer.

## 1. Introduction

The use of deep neural networks (DNN) in different scenarios related to Machine Learning (ML) applications has developed in such a way that currently neural network designs have billions of parameters with a great capability of prediction, as one of the most used types of architecture in prediction tasks. Specifically, some of those applications include image, sound, and textual data recognition. In contrast to other ML algorithms, the DNNs have achieved a remarkable accuracy. However, the use of these networks in memory and processing resource constrained devices is limited due to the amount of data needed to develop these architectures and the high computation costs for training them. Consequently, different reduction techniques are essential to fit these former networks in resource constrained devices, such as edge devices.

Among others, the most used and effective way to shrink these networks is the use of techniques such as pruning and quantization. The former one consists of removing parameters (neurons or weights) that have negligible contribution while maintaining the accuracy of the classifier. On the other hand, quantization involves replacing datatypes to reduced width datatypes, by transforming data to fit into new datatypes’ shapes. In this way, reduced networks are able to compete with the original ones in terms of accuracy and even improve these in some cases in which overfitting issues were hindering their predictability. Moreover, by reducing the width of the data, edge devices could face the storage issue mentioned above and collect larger datasets in constrained memory sizes.

Mainly convolutional neural networks (CNN) became a widely used network structure in image recognition tasks. Such a success is built upon a large number of model parameters and convolutional operations. As a result, the huge storage and computation costs make these models difficult to be deployed on resource-constrained devices, such as phones and robots, needing to adopt different reduction techniques.

In this work, we introduce a new variant to the Sparse Low Rank (SLR) method to develop weight pruning in well-known architectures, SLRProp. We judge that the last Fully Connected (FC) Layer, Final Response Layer (FRL), is the most relevant to the final decision. Moreover, the relevance of weights of this final layer are propagated to the previous layers, making each neuron non-independent of the previous layers in terms of relevance. Consequently, the connections of each neuron has a direct relationship with neuron’s predictability in the final decision of the network, needing to consider them. After factorizing the weight matrices of FC Layers, we sparsified them only considering the most relevant parts and propagate these relevances to the previous FC layers by considering the connections between different FC layers. Similarly, we performed a parallel process in which the sparsification of matrices has been carried out independently between layers, only considering the relevance intra-layer. Finally, we state the validity of the supposition of backpropagating the relevance within layers. As a result, the pruning process is optimized by determining the less relevant components of each layer, as a consequence of the addition of the backpropagation concept to the Sparse Low Rank Method contributed in this work.

### State of the Art

There have been several attempts to reduce DNNs dimensionality by applying the techniques mentioned above. Pruning techniques consist of removing part of connections (weights) or neurons from the original network so as to reduce the dimension of the original structure by maintaining its ability to predict. The core of these techniques reside on the redundancy that some elements add to the entire architecture. Memory size and bandwidth reduction are addressed with these techniques. Redundancy is lowered and overfitting is faced in some scenarios. Different classifications of works based on this ability are made depending on element pruned, structured/unstructured (symmetry), and static/dynamic.

Static pruning is the process of removing elements of a network structure offline before training and inference processes. During these last processes no changes are made to the network previously modified. However, removal of different components of the architecture requires a fine-tuning or retraining of the pruned network. This is a direct consequence of the changes that suffer the network by removing big part of its elements. Thus, some computation effort is needed in order to reach comparable accuracy to the original network.

The pruning has been carried out by following different criteria. In [1,2], they used the second derivative of the Hessian matrix to reduce the dimension of the original architecture. Optimal Brain Damage (OBD) and Optimal Brain Surgeon (OBS) work under three assumptions. Quadratic: the cost function is near quadratic. Extremal: the pruning is conducted after the network converged. Diagonal: sums up the error of individual weights by pruning the result of the error caused by their co-consequence. Additionally, OBS avoids the diagonal assumption and improves neuron removal precision by up to 90% reduction in weights for XOR networks. Using Taylor expansions of first order [3,4] was also an alternative to the previous ones to tackle networks’ dimension issues, as a criterion to approximate the change of loss in the objective function as an effect of pruning.

Some works were based on the magnitude of the elements themselves. It is undoubtedly true that near-zero values of weights make far less contributions to the results than others that surpass a certain threshold value. In this way, removing connections that may appear unnecessary, the original network is shrunk. The best way to accomplish this is the removal of weights layer by layer to not abruptly decrease the performance of the resulting network. LASSO [5] was introduced as a penalty term. It shrinks the least absolute valued feature’s corresponding weights by increasing weight sparsity. This operation has been shown to offer a better performance than traditional procedures such as OBS by selecting the most significantly contributed variables instead of using all the variables, achieving approximately 60% more sparsity than OBS. The problem with LASSO is that is an elementwise pruning technique leading to an unstructured network and sparse weight matrices. By performing this technique dividing the process by groups—as Group LASSO [6] does, removing entire groups of neurons and maintaining the original network’s structure—this last issue was solved. Groups are made based on geometry, computational complexity, or group sparsity, among others.

Singular Value Decomposition (SVD) is an effective and promising technique to shred convolutional or FC layers by reducing the number of parameters needed to represent them. Not only it has been useful for image classification tasks, but also in object detection [7] scenarios and others related with DNN-based acoustic modeling [8,9]. Low-rank decomposition for convolution layers as well as fully connected layers were applied in several works. Kholiavchenko et al. [10] proposed an iterative approach to low-rank decomposition by applying dynamic rank selection to image classification and object detection models. One of its negative aspects was that iteratively applying low-rank decomposition needs longer time and higher computational resources for rank selection in deeper models. The alternative proposed by [11] assumes the properties of both low-rank and sparseness of weight matrices while aiming to reconstruct the original matrix. In [12], through mixing the concepts of sparsity and existence of unequal contributions of neurons towards achieving the target, the Sparse Low Rank (SLR) method is proposed—a method that scatters SVD matrices to compress them by conserving lower rank for unimportant neurons. As a result, it is feasible to reduce the 3.6× storage space of SVD without much variance on the model accuracy. Speedup in the computation was another advantage that has the structured sparsity obtained by the presented approach.

The majority of the previous works had paid attention to the individual pruning of layers while not considering the connection between different layers. In [13], they claimed that the last FC layer is the most relevant of the entire network regarding the effect on the final response of the entire network. Considering this last, they proposed to prune the previous layer of the network when considering the connections of neurons with the neurons of this last FC layer called the Final Response Layer (FRL). In this way, the relevances of the neurons considered independently for the FRL were backpropagated to the previous layer’s neurons. The pruning of the rest of the layers was carried out similarly, scoring the relevance of the neurons when considering the connections with the posterior layers’ neurons.

Other alternatives have been proposed to carry out static pruning. In [14], they proposed an innovative method for CNNs pruning called layerwise relevance propagation. Each unit’s relevance to the final decision is measured, and the units that are below a predefined threshold are removed from the original structure. As a last step, each component’s relevance is recalculated by calculating the total relevance per layer to keep it constant through the iterations. Thus, each unit’s relevance is recalculated to maintain this value. In [15], a method of pruning redundant features along with their related feature maps, according to their relative cosine distances in the feature space, is proposed, and the authors achieve smaller networks with a significant download in post-training inference computational costs and achieving a decent performance. Redundancy can be minimized while inference cost (FLOPS) is reduced by 40% for VGG-16, 28%/39% for ResNet-56/110 models trained on CIFAR-10, and 28% for ResNet-34 trained on ImageNet database with almost negligible loss of accuracy. To fix the decrease in accuracy after pruning, models were retrained for some iterations maintaining all hyper-parameters.

## 2. Material and Methods

In this section, we describe the methodology proposed in order to attempt to improve the results obtained in the literature for different neural networks and datasets. Additionally, we present the datasets and models used for experimentation.

### 2.1. Methodology

The approach we present in this study follows this methodology. First, traditional low-rank decomposition SVD is applied to the weight matrix of the final FC layer, called FRL. Next, input and output weights in the layer are selected for sparsification using different neuron selection strategies. Then, sparsification is applied to the selected input and output neuron components in the decomposed matrices. With the most relevant neurons of the final FC layer obtained we back propagate their relevance to the prior FC layer, following the idea proposed by [13], and we obtained the relevance of the neurons composing the prior FC layer. Finally, we repeated the process of sparsification for the decomposed matrices of the prior FC layer. In parallel, we performed the same process of sparsification but only considering the relevance of each individual layer for the last two FC layers. The results and comparative of both methodologies are summarized in Section 4.

#### 2.1.1. Single Value Decomposition (SVD)

One way for decomposing matrices representing the weights of neural networks is the use of low-rank factorization. A convolutional neural network is composed of a large number of convolutional layers and fully connected layers. By applying this technique to convolutional kernels weights optimization of the inference speed, the convolution operation could be obtained due to the reduction in the time needed for multiplication with factorized matrices compared to that of multiplication with 3D weights of kernels.

In a FC layer having m input and n output neurons, activation $a\in R^{n}$ of the layer with n nodes is represented as

(1) $$a=g(W^{T}X+b)$$

where X represents the input to the layer, and **g**() represents any of the possible activation functions. FC layers connections form a weight matrix $W\in R^{m\times n}$ and a bias vector $b\in R^{n}$ where each parameter in the weight matrix W is $w^{ij}\in R$(1≤i≤m,1≤j≤n), and bias matrix b is $b^{j}\in R\left(1\leq j\leq n\right)$. The proposed approach is applied to the weight matrix W after training the entire model. The SVD approach decomposes the weight matrix W as $W=USV^{T}$ where $U\in R^{m\times m}$, $V^{T}\in R^{n\times n}$ are orthogonal matrices and $S\in R^{m\times n}$ is a diagonal matrix.

#### 2.1.2. Sparse Low Rank Decomposition

The matrix S is a diagonal matrix containing n non-negative singular values in a decreasing order. The *k* singular values that are most significant are kept by Truncated SVD where the decomposed matrices U,S, and VT become $\hat{U},\hat{S},{\hat{V}}^{T}\in R^{m\times k},R^{k\times k},R^{k\times n}$. By this way, the original weights W are replaced into reconstructed approximated weight $\hat{W}$ as $\hat{W}=\hat{U}\hat{S}{\hat{V}}^{T}$.

In SVD we have diagonal matrix sigma S with the most significant singular values from the upper left to lower right in a decreasing order. In the truncation process the first k rows of U and columns of ${\hat{V}}^{T}$ are kept.

Simulating the approach driven by [12] we compressed truncated matrices $\hat{U},\hat{S}$, and $\hat{V}$ based on the importance of the m input and n output neurons, i.e., we represented a few columns of $\hat{U}$ and rows of ${\hat{V}}^{T}$ with a rank lower than *k*, called reduced rank rk. In this way, only rk most significant rows and columns are kept in $\hat{U}$ and ${\hat{V}}^{T}$, respectively, due to the order of importance of W that starts from left to right through columns of $\hat{U}$ and top to bottom through rows of ${\hat{V}}^{T}$. We considered only the most significant rows (rm) and columns (rn) from each column and row from $\hat{U}$ and ${\hat{V}}^{T}$, respectively, following the cost criteria, briefly explained in the next subsection.

When the matrices $\hat{U},\hat{S}$ and ${\hat{V}}^{T}$ are sparsified with sr and rr, the total number of non-zero parameters of the $\hat{U},\hat{S},{\hat{V}}^{T}$ become k(m−rm+n−rn+1)+rk(rm+rn), which is less than the number of non-zero parameters of truncated SVD k(m+n+1).

Pruning fully connected layers is much more effective in terms of accuracy, time, and energy efficiency than pruning convolutional layers as shown in [16], which contributes to bigger losses in prediction capability with the same rate of reduction in parameters. Those are usually placed in the first positions in DNNs, and they are more sensitive than the ones that are placed in the last positions in many cases. In this study, we followed the approach directed by [12] sparsifying SVD matrices achieving a low compression rate without big losses in accuracy. We used as a metric of sparsification the compression rate defined in [12], as the ratio between the parameters needed to define the sparsified decomposed matrices and the original weights’ matrix parameters. In our case, we analyzed their 3 variants of applying SLR, that were based in cost, weights, and activations, and we proposed two new variants that sum the importance of cost and weights and cost and activations due to the fact that each of them performed as the best variant in different compression rate regimes.

Overall, the most relevant attribute was the cost, so we decided to establish this as the criteria for selection of the rows and columns for sparsification. An explanation of the full process of this method is given in Algorithm 1.
**Algorithm 1** SLRPropWeights1←FRL weightsWeights2←Previous FC layer weightsU,S,V←SVD(Weights1)tU,tS,tV←U[:,0:rank],S[0:rank],V[0:rank,:]**for**Nrows**do**    tempU[row,rk:]=0    Weightst←tempU∗tS∗tV    Score←Accuracy(Weightst)    is←Ranking of rows**end for****for**Ncolumns**do**    tempV[rk:,column]=0    Weightst←tU∗tS∗tempV    Score←Accuracy(Weightst)    os←Ranking of columns**end for**U(rmrows)←0 where rm=sr*mV(rncolumns)←0 where rn=sr*nU2,S2,V2←SVD(Weights2)tU2,tS2,tV2←U2[:,0:rank],S2[0:rank],V2[0:rank,:]**for**Nrows**do**    Score←∑Abs(U2[i,j]∗is[j])    is2←Ranking of rows**end for****for**Ncolumns**do**    tempV2[rr:,column]=0    Weightst2←tU2∗tS2∗tempV2    Score←Accuracy(Weightst2)    os2←Ranking of columns**end for**U2(rmrows)←0V2(rmcolumns)←0

#### 2.1.3. Selection of Rows and Columns Based on Cost

A neuron’s importance is defined by whether there is a change or not in the network performance after removing it. Let *c* be the default cost of the neural network with original trained weight *W* estimated for the p training samples, computed using any loss function. Let $\hat{c}$ be the value of cost of the network with sparsified weights $\hat{W}$. By truncating with reduced rank rr a specific row of $\hat{U}$ or column of ${\hat{V}}^{T}$ we have the absolute change in cost is or os. Those are calculated as follows:

(2) $$is_{i}=\left|c-{\hat{c}}_{i}\right|$$

(3) $$os_{j}=\left|c-{\hat{c}}_{j}\right|$$

As the sparsification process purpose is to ensure that the functionality of the network does not change after compression, and not to reduce the overall network cost or improve accuracy, only the absolute change in the cost value is considered.

#### 2.1.4. Propagation of Relevance between Layers

As it is known, the majority of neural networks can be formulated as a nested function. Thus, we can define a network with *n* hidden layers as a $F^{\left(n\right)}=f^{\left(n\right)}\circ f^{\left(n-1\right)}\circ ...\circ f^{\left(1\right)}$. Each layer can be represented as follows:

(4) $$f^{\left(n\right)}\left(x\right)=\sigma^{\left(n\right)}\left(w^{\left(n\right)}x+b^{\left(n\right)}\right)$$

where $\sigma^{\left(n\right)}$ is the activation function of each layer, $w^{\left(n\right)}$ is the corresponding layers connections’ weight function, and $b^{\left(n\right)}$ is the bias of each layer. At this stage it is possible to say that all of these layers are interconnected and each of them has direct relevance on the final decision of the entire network. Consequently, weights from the FRL, that is the last Fully Connected Layer, backpropagate their relevance to the prior layers as proposed in [13]. As a result, the relevance of each neuron in the final decision is the composition of weights that are interconnected until the FRL corresponding element’s relevance. The summation of the corresponding relevances is given by Equation (5).

(5) $$s_{k}=|w^{\left(k+1\right)}{|}^{⊤}|w^{\left(k+2\right)}{|}^{⊤}...{\left|w^{\left(n\right)}\right|}^{⊤}s_{n}$$

The absolute value of the weights that are connected to each of the neurons of the FRL are multiplied by the relevance of these in the FRL.

(6) $$s_{k,j}=\sum_{i}\left|w_{i,j}^{\left(k+1\right)}\right|s_{k+1,i}$$

Equation (6) shows the relevance of the j-th neuron in the *k*-th layer, which propagates the relevances of the neurons from the posterior k+1-th layer that are connected with it.

By introducing this idea to the SVD matrices, keeping only the most relevant rows of U matrices, we can consider only the most relevant neurons of that layer. The procedure in the FC layers that are not the FRL, is similar to the original SLR method except for the sparsification of the U matrices where the relevance propagated through the posterior layers is considered to determine the most relevant neurons. This relevance is propagated following Equation (6).

In summary, the main contributions made by this work are the following. The pruning of weights carried out in these FC layers is more optimal than in the original SLR method. Consequently, the performance of the resulting network is raised, obtaining sub-optimal results in terms of different performance metrics defined in Section 4 with far less weights needed compared with the original structure. Thus, in scenarios in which original network structures cannot fit end user devices due to memory restrictions are crucial for such reduction techniques.

### 2.2. Materials

Regarding the materials, we used two well-known models for image recognition, VGG-16 [17] and Lenet5 [18], where VGG architecture is much known for its memory intensive FC layers. It is worth noting that VGG is the commonly used architecture with FC layers where other popular image recognition models, such as ResNet, Inception, MobileNet, ResNet, DenseNet, and object detection models, do not have FC layers except the final softmax layer. Table 1 and Table 2 show the specifications of each network structure.

These two different approaches were tested on different well-known datasets, Cifar10 (VGG16), Cifar100 (VGG16), and MNIST (Lenet5). Each of them contain 32 × 32 images (color images in Cifar10/Cifar100 and grayscale images in MNIST). In case of Cifar10 and MNIST there are 10 different classes and 100 in Cifar100. All of them have been trained using default 10,000 test images and 50,000 and 60,000 training images for the Cifar and MNIST datasets, respectively. Different compression rates were applied for sparsifying SVD matrices; therefore, for each dataset we obtained different performance metrics for each method. Overall, we were able to state which method was the best in each case. The datasets used for experiments comprise a good mix of different image types, sizes, and number of classes. CIFAR-10 and CIFAR-100 have general purpose image classes where MNIST dataset contains handwritten digit images.

Moreover, to demonstrate the usefulness of our approach in sensor related data we tested our approach in a model consisting of 3 FC layers for the Room Occupancy Estimation Data Set from the UCI Machine Learning Repository. It is a dataset for estimating the precise number of occupants in a room using multiple non-intrusive environmental sensors such as temperature, light, sound, ${\mathrm{CO}}_{2}$, and PIR. There are 10,129 instances using 1000 for testing and the rest for training. Table 3 shows the specifications of the network structure.

The environment in which all development of our work had been processed is a ×64 Ubuntu 20.04.4 LTS Operating System equipped with an Intel Core i7-11850H working at 2.5 GHz × 16 and 32 GB DDR-4 RAM and a NVIDIA T1200 Laptop GPU (driver version: 510.47.03, CUDA version:11.6).

## 3. Proposed Approach

As cited above, the intention of this research was to realize the connection of relevances between different layers. To do so, we opted for applying the approach presented by [12] in two different FC layers. First, we applied it independently. To show that there is a direct relationship between neurons from different layers, we considered the relevance of the FRL and backpropagate it until the second FC layer that we pruned in the parallel process. In this way, we could see the effect of backpropagating the relevance throughout layers and see the correlation between them.

We applied the SLR approach proposed by [12] to obtain information about the most relevant parts forming the FRL. In this way, we were able to know the relevances for the final decision of each of the neurons comprising this last FC layer. To calculate the relevance propagated to the previous layers we used the insight introduced in Section 2.1.4 and multiplied each of the absolute value of weights that was connected with each neuron of the next layer with the relevance of these neurons from the next layer, for each neuron comprising the layer in question. Finally, after obtaining the relevances for each neuron from the layer, we sparsified the weight matrix of this layer the same way as for the FRL but while sparsifying the U matrix in the following way. We considered only the rows that obtained the highest value after the summation of multiplications of absolute weights of connections with each of the relevances of neurons connected from the next layer, instead of considering the original relevances of neurons as we implemented for the FRL.

**Figure 1:**
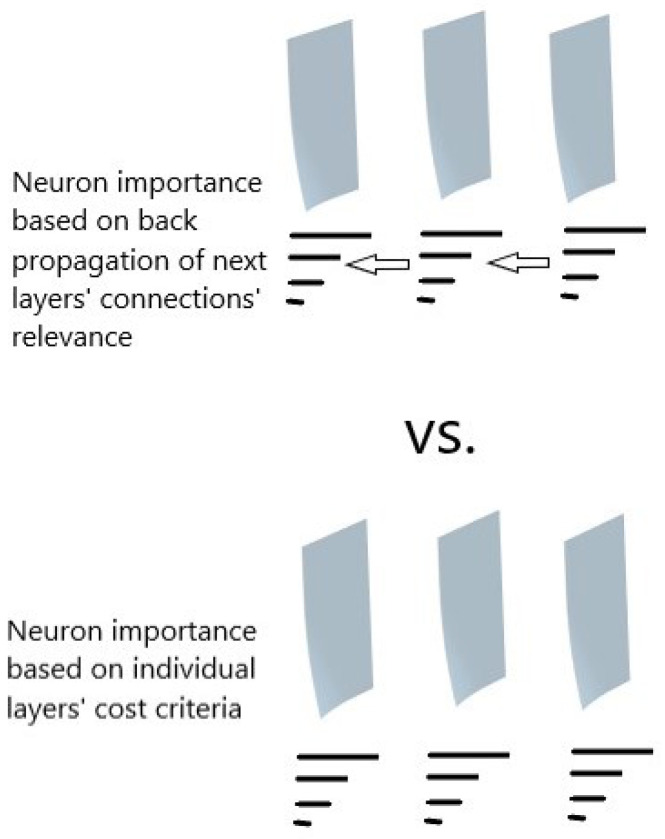
Comparison of the proposed approaches.

At the same time, we carried out sparsification of the same number of layers only considering the independent relevances of each layer, following the criteria proposed by [12]. In this work, they present three different criteria to determine which elements of each layer were more relevant to the final decision of the network. Overall, the criteria based in the cost of weights was the most adequate to reduce the dimensionality of the problem and maintain the performance of the architecture to be as high as possible. The graphical representation of both approaches is given by Figure 1.

In case of VGG16, the FRL corresponds to FC7, and the backpropagation of the relevances has been carried out until FC6. FRL and previous FC layer of Lenet5 are FC4 and FC3, respectively. In case of the aforementioned three FC layers’ architecture these layers are FC3 and FC2, respectively.

## 4. Experiment

In this section, details about the entire experimentation process are described. The results obtained are summarized as well.

### Performance Metrics

Evaluation metrics used for determining which of the methods used is best for keeping the performance of the former network as high as possible are the accuracy vs. compression rate, AUC vs. compression, recall vs. compression, precision vs. compression, and specificity vs. compression, where the compression rate was defined in [12]. This last metric determines the relationship of the number of parameters between sparsified decomposed matrices and the original network’s weight matrices. AUC is the area below the ROC curve—i.e., a graph showing the performance of a classification model at all classification thresholds. What is plotted in the curve is the FPR and TPR in the x and y axes, respectively, whose definitions are given in Equation (10) and (11). The definitions of the rest of the metrics mentioned above are given in Equations (7)–(9), where TP, TN, FP, and FP stand for True Positives, True Negatives, False Positives, and False Negatives, respectively. We used FRL’s previous FC layer’s compression rate to check the accuracy of the resultant network on different compression rate regimes.

Each of the variants proposed in this work, considering or not the relevance between layers, have been tested on well-known open source datasets for image recognition Cifar10, Cifar100, and MNIST. All of them have been trained using default 10,000 test images and 50,000 and 60,000 training images for the Cifar and MNIST datasets, respectively. To show their effectiveness in sensor related datasets, they were applied to the Room Occupancy Detection Dataset too. In this case, 1,000 samples were used for testing and the rest (9,129 samples) for training the network. In each case, we opted for establishing the same reduction rate (0.5) and sparsity rate (0.5) defined in [12], and we tested each variant with different rank k, which determines the number of columns and rows kept in the sparsified $\hat{U}$ and ${\hat{V}}^{T}$ matrices. We incremented the rank k until the performance metrics were equal to the ones obtained by the original network structure. In the testing phase 10 different seeds were established for testing each methodology in each dataset.

(7) $$Accuracy\left(Acc\right)=\frac{TP+TN}{TP+TN+FP+FN}$$

(8) $$Recall\left(Re\right)=\frac{TP}{TP+FN}$$

(9) $$Precision\left(Pr\right)=\frac{TP}{TP+FP}$$

(10) $$TruePositiveRate\left(TPR\right)=\frac{TP}{TP+FN}$$

(11) $$FalsePositiveRate\left(FPR\right)=\frac{FP}{FP+TN}$$

(12) $$Specificity\left(Spec\right)=\frac{FP}{FP+TN}$$

## 5. Results

**Figure 2:**
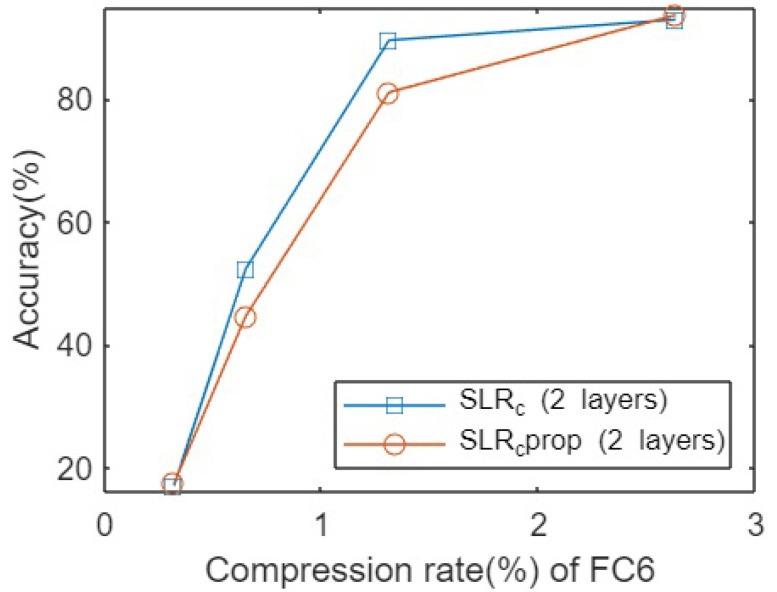
Accuracies for pruning VGG16 network on Cifar10 dataset for different compression rates.

Figure 2 shows the accuracies obtained after testing both pruning techniques for the VGG16 architecture on the Cifar10 dataset. As is clear, there was no significant difference between the methods when applying an extremely low compression rate, which means that very few parameters of the original matrices were kept. Similarly, we could observe the same pattern when a higher number of parameters were kept in the original decomposed matrices, but there were significant differences between both compression rate regimes. In this case, applying the SLR method independently to different FC layers offers a higher accuracy with the same compression rate, i.e., keeping the same number of connections between neurons.

**Figure 3:**
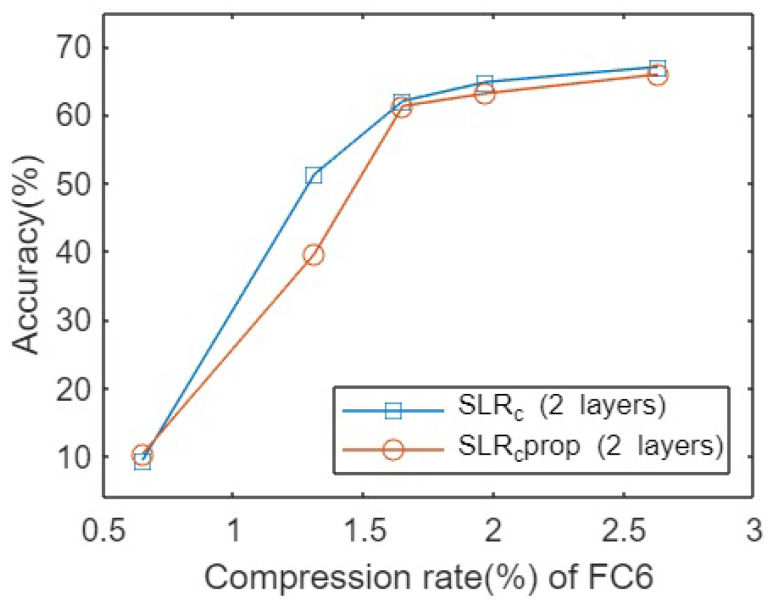
Accuracies for pruning VGG16 network on Cifar100 dataset for different compression rates.

Similarly, there could be the same pattern in case of Cifar100 dataset for the same network architecture. The SLR method was applied independently without any consideration of propagation of relevances across layers. In this case for lower compression rate regimes the difference is high as well. Figure 3 shows the results summarized for the Cifar100 dataset.

**Figure 4:**
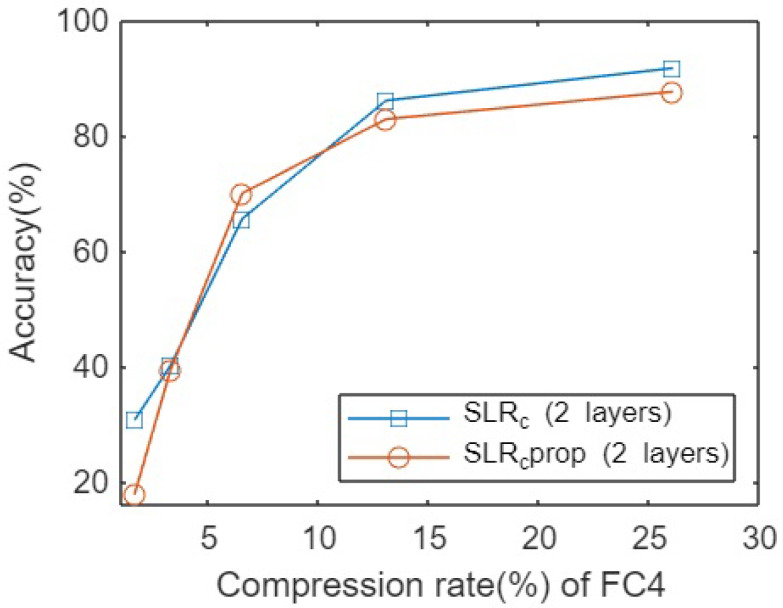
Accuracies for pruning Lenet5 network on MNIST dataset for different compression rates.

Simultaneously, Lenet5 architecture was pruned following both methodologies on MNIST dataset. Figure 4 shows the accuracies obtained for different compression rates. As could be observed, for the majority of the pruning rates applied when applying the SLR independently in different layers offers better performance than considering the backpropagation rule of the relevances from the FRL. However, in certain compression rate regimes, the last one outperforms the former one, but the difference is insignificant compared to the overall performance result.

**Figure 5:**
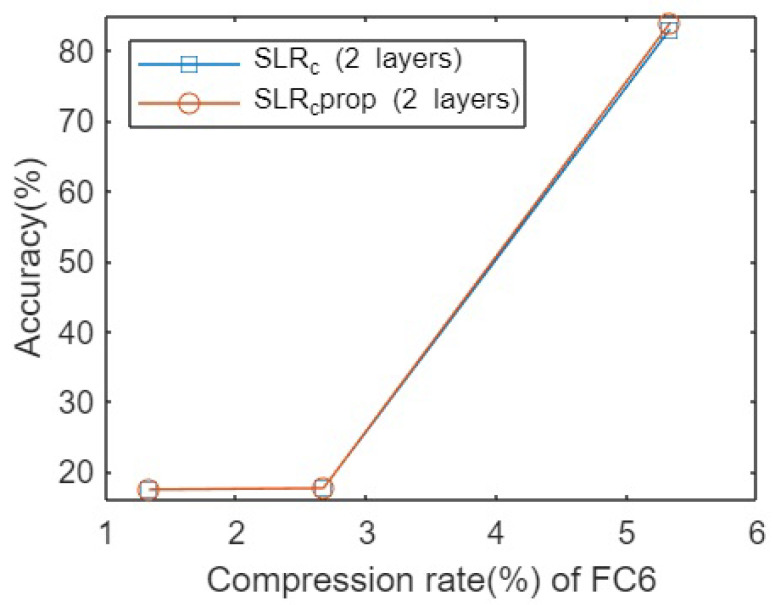
Accuracies for pruning FC layers architecture on Room Occupancy Detection dataset for different compression rates.

Finally, both methodologies for pruning FC layers were adopted for pruning FRL and the previous FC layer of the Room Occupancy Detection dataset. Figure 5 shows the accuracies obtained for different compression rates. There is a clear tendency towards SLRProp in terms of accuracy, which determines that for the majority of the regions of compression SLRProp outperforms the SLR method.

Overall, if we observe in detail each of the metrics defined in the previous section and given in Appendix A, we can obtain a general verdict about the performance of SLR and SLRProp applied to the four datasets described in Section 2. In 30 cases SLR obtained a higher metric value, and in 33 cases SLRProp obtained a better performance result. In 14 cases the results are identical for both methods. In Table 4 a brief review of these metrics is given. Once having this comparison, we can deduce that SLRProp’s performance is slightly better than the original SLR that was presented by [12].

## 6. Discussion

As demonstrated in the previous section, the introduction of the concept of backpropagation of the relevances from the FRL to the rest of the layers of the original network does not always outperform the supposition of the relevances independently within layers. However, the general result shows that the SLRProp method is slightly better than the original version of sparsification presented by [12]. In this way, the breakthrough presented by [13] is preserved in this experiment; the final result the relevances propagated between different layers through the connections between neurons is of particular importance. For relatively high compression rate regimes, where the number of pruned connections is not very high, the performance metrics are almost identical for all architectures applied for the four different datasets. On the contrary, for very low compression rate regimes the performance metrics do not follow a distinguishable pattern, which shows the randomness of both methods when an excessive pruning is carried out in any of the mentioned architectures.

This shows that the components of each layer have certain influence on the rest of the network components, even though the main contribution to the final result of each component is more connected with other aspects than the connections’ weights’ absolute values across layers. In this case, the cost defined as the difference of the accuracy between the case when a certain component is eliminated from the original network and the original structure’s accuracy showed that it could be more crucial when selecting which connections should be removed when pruning the original network. Additionally, backpropagating the relevances of the FRL to the previous FC layers could yield an even more adequate performance when applied to certain datasets, e.g., for the Room Occupancy Detection dataset. Consequently, this paper shows that the relevances propagated between layers play an important role when determining which are the most important components of the network structure.

To summarize, it is possible to say that the proposition presented by [13] echoed in Equation (6) is conserved in this experimental process, thus challenging its validity for every architecture of a convolutional neural network focused on image recognition. In fact, the ranking of the connection’s relevance proposed by [12] offers an optimal result in terms of accuracy and network compression—needing only a very low percentage of parameters for representing sparsified matrices compared to the original network’s matrices. However, the computational cost of calculating each matrices’ components costs might be too high and ineffective in many scenarios, which creates the need for an alternative method for solving this issue of the training phase. The SLRProp alternative offers a slight improvement in different accuracy metrics, but is still too costly in terms of time efficiency and computational load. Attending these networks’ weights’ absolute values as a criteria to decide which columns and rows are maintained in the sparsified matrices offers a near identical result in terms of accuracy that needs ∼100x less time for sparsifying the SVD matrices in the training phase. In applications where time response is crucial, this last alternative method may be more adequate.

## Figures and Tables

**Table 1:** VGG16 model trained for 32 × 32 images.

Layer Name	Layer Type	Feature Map	Output Size of Images	Kernel Size	Stride	Activation
Input	Image	1	32 × 32 × 3	-	-	-
Conv-1	2 × Conv	64	32 × 32 × 64	3 × 3	1	relu
Pool1	Maxpool	64	16 × 16 × 64	3 × 3	2	relu
Conv-2	2 × Conv	128	16 × 16 × 128	3 × 3	1	relu
Pool2	Maxpool	128	8 × 8 × 128	3 × 3	2	relu
Conv-3	2 × Conv	256	8 × 8 × 256	3 × 3	1	relu
Pool3	Maxpool	256	4 × 4 × 256	3 × 3	2	relu
Conv-4	2 × Conv	512	4 × 4 × 512	3 × 3	1	relu
Pool4	Maxpool	512	2 × 2 × 512	3 × 3	2	relu
Conv-5	2 × Conv	512	2 × 2 × 512	3 × 3	1	relu
Pool5	Maxpool	512	1 × 1 × 512	3 × 3	2	relu
Flatten	Flatten	-	512	-	-	relu
FC6	Dense	-	4096	-	-	relu
FC7	Dense	-	4096	-	-	relu
FC8	Dense	-	# of classes	-	-	softmax

**Table 2:** Lenet5 model trained for 32 × 32 images.

Layer Name	Layer Type	Feature Map	Output Size of Images	Kernel Size	Stride	Activation
Input	Image	1	32 × 32 × 3	-	-	-
Conv-1	1 × Conv	6	28 × 28 × 6	5 × 5	1	tanh
Pool1	Avgppool	6	14 × 14 × 6	2 × 2	2	tanh
Conv-2	1 × Conv	16	10 × 10 × 16	5 × 5	1	tanh
Pool2	Avgppool	16	5 × 5 × 16	2 × 2	2	tanh
Flatten	Flatten	-	400	-	-	tanh
FC3	Dense	-	120	-	-	relu
FC4	Dense	-	84	-	-	relu
FC5	Dense	-	# of classes	-	-	softmax

**Table 3:** FC layers model.

Layer Name	Layer Type	Output Size	Activation
Input	Data	# of attributes	-
FC1	Dense	4000	relu
FC2	Dense	4000	relu
FC3	Dense	4000	relu
FC4	Dense	# of classes	sigmoid

**Table 4:** SLR vs. SLRProp accuracies for different datasets.

Rank k	Cifar10-SLR	Cifar10-SLRProp	Cifar100-SLR	Cifar100-SLRProp	MNIST-SLR	MNIST-SLRProp	Room-SLR	Room-SLRProp
k = 2	17.037%	17.4074%	4%	2.8519%	21.084%	23.492%	17.5%	17.5
k = 4	52.3333%	43.5185%	9.8889%	8.8889%	38.006%	40.053%	17.7%	17.7%
k = 8	89.5926%	81.4444%	47.4444%	37.9259%	71.597%	80.469%	83%	83.9%
k = 16	92.963%	92.963%	63.4074%	62.4444%	93.258%	94.245%	-	-
k = 32	-	-	-	-	98.416%	98.385%	-	-

## Data Availability

The raw data used in this work is available at https://keras.io/api/datasets/ (accessed on 12 December 2022) and at https://archive.ics.uci.edu/ml/index.php (accessed on 16 December 2022) were a brief explanation of each dataset is given, as well as and explanation of how to use the data.

## Sample Availability

Samples of the compounds are available from the authors.

## Abbreviations

The following abbreviations are used in this manuscript:

AUC	Area Under the ROC Curve
CNN	Convolutional Neural Networks
DNN	Deep Neural Networks
FC	Fully Connected
FN	False Negatives
FP	False Positives
FPR	False Positive Rate
FRL	Final Response Layer
ML	Machine Learning
OBD	Optimal Brain Damage
OBS	Optimal Brain Surgeon
SLR	Sparse Low Rank Method
SVD	Singular Value Decomposition
TN	True Negatives
TP	True Positives
TPR	True Positive Rate

## Appendix A

In this appendix the performance metrics described in this article for each of the datasets mentioned in Section 2 are given.

**Table A1:** SLR vs. SLRProp Cifar10.

Method-Rank k	Accuracy	AUC	Recall	Precision	Specificity
SLR-k = 2	17.037%	52.2763%	0%	0%	-
SLRProp-k = 2	17.4074%	50.6373%	0%	0%	-
SLR-k = 4	52.3333%	77.6934%	80.9155%	26.9259%	-
SLRProp-k = 4	43.5185	74.7603	62.4146%	17.4074%	-
SLR-k = 8	89.5926%	98.7243%	86%	92.0401%	-
SLRProp-k = 8	81.4444%	97.3784%	74.037%	87.3035%	-
SLR-k = 16	92.963%	99.1493%	92.9630%	93.2764%	-
SLRProp-k = 16	92.963%	99.1873%	92.9630%	93.5727%	-

**Table A2:** SLR vs. SLRProp Cifar100.

Method-Rank k	Accuracy	AUC	Recall	Precision	Specificity
SLR-k = 2	4%	68.0924%	0%	0%	100%
SLRProp-k = 2	2.8519%	65.6321%	0%	0%	100%
SLR-k = 4	9.8889%	80.7851%	0.0741%	0.6667%	99.9996%
SLRProp-k = 4	8.8889%	81.2581%	0.1481%	4%	100%
SLR-k = 8	47.4444%	96.5314%	18.3333%	86.0334%	99.9719%
SLRProp-k = 8	37.9259%	95.0432%	12.6667%	77.8508%	99.9637%
SLR-k = 16	63.4074%	96.2422%	54.7037%	76.9269%	99.8316%
SLRProp-k = 16	62.4444%	96.2004%	53.0741%	79.3686%	99.8578%

**Table A3:** SLR vs. SLRProp MNIST.

Method-Rank k	Accuracy	AUC	Recall	Precision	Specificity
SLR-k = 2	21.084%	58.7361%	20.914%	21.176%	91.3463%
SLRProp-k = 2	23.492%	59.857%	23.309%	23.5553%	91.5947%
SLR-k = 4	38.006%	74.2785%	37.41%	38.4775%	93.3527%
SLRProp-k = 4	40.053%	77.2657%	39.267%	40.6033%	93.6218%
SLR-k = 8	80.469%	94.7351%	80.219%	80.774%	97.8786%
SLRProp-k = 8	71.597%	92.8456%	70.98%	72.2031%	96.3698%
SLR-k = 16	93.258%	98.6997%	93.16%	93.3691%	99.2649%
SLRProp-k = 16	94.245%	98.856%	94.167%	94.3613%	99.3748%
SLR-k = 32	98.416%	99.7014%	98.393%	98.455%	99.8284%
SLRProp-k = 32	98.385%	99.7007%	98.366%	98.43%	99.8257%

**Table A4:** SLR vs. SLRProp Room Occupancy Estimation.

Method-Rank k	Accuracy	AUC	Recall	Precision	Specificity
SLR-k = 2	17.5%	49.8478%	17.5%	17.5%	72.5%
SLRProp-k = 2	17.5%	49.9085%	17.5%	17.5449%	72.5833%
SLR-k = 4	17.7%	62.6004%	17.5%	19.774%	76.3333%
SLRProp-k = 4	17.7%	63.3562%	17.5%	19.774%	76.3333%
SLR-k = 8	83%	93.9923%	49.8%	73.5598%	94.0333%
SLRProp-k = 8	83.89%	95.5216%	51.8%	77.54449%	95.08%
